# The genetic diversity of cereulide biosynthesis gene cluster indicates a composite transposon Tn*ces* in emetic *Bacillus weihenstephanensis*

**DOI:** 10.1186/1471-2180-14-149

**Published:** 2014-06-07

**Authors:** Xiaofen Mei, Kai Xu, Lingling Yang, Zhiming Yuan, Jacques Mahillon, Xiaomin Hu

**Affiliations:** 1 Key Laboratory of Agricultural and Environmental Microbiology, Wuhan Institute of Virology, Chinese Academy of Sciences, Wuhan 430071, China; 2 University of the Chinese Academy of Sciences, Beijing 100039, China; 3 Laboratory of Food and Environmental Microbiology, Université catholique de Louvain, Croix du Sud 2 - L7.05.12, Louvain-la-Neuve B-1348, Belgium

**Keywords:** Cereulide, *Bacillus cereus*, *Bacillus weihenstephanensis*, Transposon, Plasmid

## Abstract

**Background:**

Cereulide is a cyclic dodecadepsipeptide ionophore, produced via non-ribosomal peptide synthetases (NRPS), which in rare cases can lead to human death. Early studies had shown that emetic toxin formation belongs to a homogeneous group of *Bacillus cereus sensu stricto* and the genetic determinants of cereulide (a 24-kb gene cluster of *cesHPTABCD*) are located on a 270-kb plasmid related to the *Bacillus anthracis* virulence plasmid pXO1.

**Results:**

The whole genome sequences from seven emetic isolates, including two *B. cereus sensu stricto* and five *Bacillus weihenstephanensis* strains, were compared, and their inside and adjacent DNA sequences of the cereulide biosynthesis gene clusters were analyzed. The sequence diversity was observed, which classified the seven emetic isolates into three clades. Different genomic locations of the cereulide biosynthesis gene clusters, plasmid-borne and chromosome-borne, were also found. Potential mobile genetic elements (MGEs) were identified in the flanking sequences of the *ces* gene cluster in all three types. The most striking observation was the identification of a putative composite transposon, Tn*ces*, consisting of two copies of IS*ces* element (belonging to IS*6* family) in opposite orientations flanking the *ces* gene cluster in emetic *B. weihenstephanensis*. The mobility of this element was tested by replacing the *ces* gene cluster by a Km^R^ gene marker and performing mating-out transposition assays in *Escherichia coli*. The results showed that Tn*ces::km* transposes efficiently (1.04 × 10^-3^ T/R) and produces 8-bp direct repeat (DR) at the insertion sites*.*

**Conclusions:**

Cereulide biosynthesis gene clusters display sequence diversity, different genomic locations and association with MGEs, in which the transposition capacity of a resistant derivative of the composite transposon Tn*ces* in *E. coli* was demonstrated. Further study is needed to look for appropriate genetic tools to analysis the transposition of Tn*ces* in *Bacillus* spp. and the dynamics of other MGEs flanking the *ces* gene clusters.

## Background

The *Bacillus cereus* group consists of *B. cereus sensu stricto*, *Bacillus thuringiensis*, *Bacillus anthracis*, *Bacillus weihenstephanensis*, *Bacillus mycoides, Bacillus pseudomycoides* and *Bacillus cytotoxicus,* which share close genetic and biochemical relatedness. They have traditionally been classified as different species based on their distinct virulence characteristics or phenotypes [1,2], the formers are mostly directly associated with large plasmids. *B. anthracis* causes the fatal animal and human disease anthrax, genetically determined by its pXO1 and pXO2 plasmids [3]. Similarly, the biopesticidal properties of *B. thuringiensis*, which distinguish it from *B. cereus*, are due to large plasmids encoding *cry* genes [4]. Ubiquitous in natural environment and best known as an opportunistic pathogen and food contaminant, *B. cereus sensu stricto* can cause two distinct forms of food poisoning with symptoms of diarrhea or vomiting. The diarrheal type, generally mild and mostly self-healed, is caused by several potential heat-labile enterotoxins, *e.g.* Hbl, Nhe, and CytK, whereas the emetic type, which represents the most serious food safety risk linked to *B. cereus*, is associated with a heat stable peptide toxin named cereulide. Most virulence genes of *B. cereus* are located on the chromosome [5,6] with the exception of the cereulide genetic determinants [7,8]. *B. cytotoxicus* is a recently described thermotolerant member of the *B. cereus* group [1]. The remaining members of the group, *B. mycoides, B. pseudomycoides* and *B. weihenstephanensis,* are mainly distinguished on the basis of their morphology (rhizoidal growth) and physiology (psychrotolerance), respectively [9,10], but may also have enteropathogenic potential [11,12]. In this respect, two *B. weihenstephanensis* isolates were found to produce a higher amount of cereulide than the reference *B. cereus* AH187 quantified by liquid chromatography mass spectrometry [13,14].

Cereulide ((D-O-Leu-D-Ala-L-O-Val-L-Val)_3_) is a small, heat and acid stable cyclic dodecadepsipeptide with a molecular weight of 1.2 kDa [15,16] and presents similar characteristics to valinomycin, *i.e.* chemical structure and toxicology [17,18]. Like valinomycin, cereulide is synthesized enzymatically via non-ribosomal peptide synthetases (NRPS), and is toxic to mitochondria by acting as a potassium ionophore [19]. It has been reported to inhibit human natural killer cells [20]. Indeed, severe and even lethal cases have been reported after the ingestion of food contaminated with high amounts of cereulide [21-24].

The cereulide genetic determinants correspond to a cluster of seven NRPS genes (*cesA, B, C, D, H, P* and *T*), which was originally found residing on a large plasmid [8]. This 270 kb element, pCER270, displays similarity to the anthrax virulence pXO1 from *B. anthracis*[7,25]. It is a member of pXO1-like plasmids, including pCER270, pPER272, pBC10987 and pBCXO1, which share a highly conserved core region containing genes involved in plasmid replication and its maintenance, sporulation and germination, and a formaldehyde-detoxification locus [25,26].

Previous studies have shown that enterotoxin production is broadly distributed among different members of the *B. cereus* group [6,27] and also found in other *Bacillus* spp. [28,29], whereas emetic toxin formation has been reported to be restricted to a homogeneous group of *B. cereus sensu strict*[30]. Although seldom, cereulide-producing *B. weihenstephanensis* strains have also recently been isolated [14]. In order to explore the phylogenetic relationship of the emetic isolates between *B. cereus sensu stricto* and *B. weihenstephanensis,* and to analyze the potential mode of genomic transfer of the cereulide genetic determinants, the genetic diversity between *B. cereus sensu stricto* and *B. weihenstephanensis* were analyzed in detail.

## Results

### Genome sequences comparison of emetic isolates

**Figure 1:**
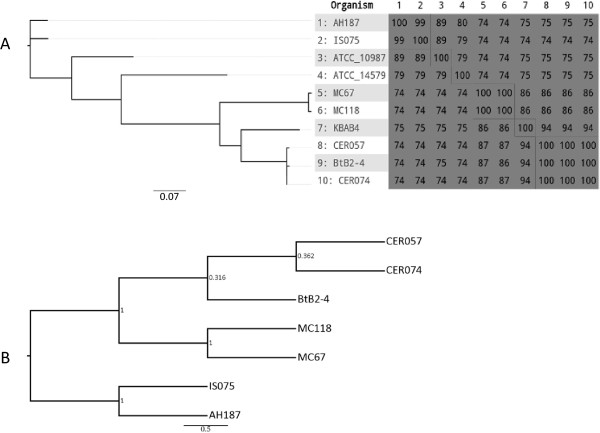
**Phylogenetic analysis based on the sequences of genomes and *ces *genes of *B. cereus *group strains. (A)** Phylogenetic overview in Gegenees of the genomes. The scale bar represents a 7% difference in average BLASTN score similarity. The heat-map is asymmetric because the variable contents of genomes differ in sizes and a similarity is calculated as a fraction of similar sequences in each genome. **(B)** Dendrogram based on the seven concatenated *ces* gene sequences by an NJ phylogenetic tree with a bootstrap of 1,000.

The comparison of 10 genome sequences including seven emetic (Table 1) and three non-emetic *B. cereus* group isolates was performed by Gegenees [31]. According to the heatmap (Figure 1A), the two emetic *B. cereus sensu stricto* isolates IS075 and AH187 show a similarity of more than 99%; and the five emetic *B. weihenstephanensis* isolates show similarities ranging from 86% to 100%, in which the similarity between MC67 and MC118, or between CER057, CER074 and BtB2-4, respectively, is 100%, whereas between MC67/MC118 and CER057/BtB2-4/CER074 is ca. 86%. Thus IS075 and AH187 share very similar gene content to form a clade in the phylogenetic tree, so do MC67 and MC118, and CER057 and CER074 and BtB2-4, respectively. CER057/BtB2-4/CER074 is more similar to *B. weihenstephanensis* KBAB4 than MC67/MC118, with similarities 94% *vs.* 86%.

**Table 1:** Emetic strains used in this study

**Strain**	**Relevant characteristics**	**Reference**	**Genome accession no. in GenBank**	**Contig containing *ces *** **gene cluster**	
				**Accession no. in GenBank**	**Length (bp)**
AH187	*B. cereus,* reference strain, containing pCER270 with the *ces* gene cluster	(7)	NC_010924	NC_010924	270,082
IS075	*B. cereus, i*solated from mammal in Poland	(13)	AHCH01000000	AHCH02000031	180,702
BtB2-4	*B. weihenstephanensis,* isolated from soil in Belgium	(13)	AHDR01000000	AHDR01000022	286,458
CER057	*B. weihenstephanensis,* isolated from parsley in Belgium	(13)	AHDS01000000	AHDS01000024	245,438
CER074	*B. weihenstephanensis,* isolated from milk in Belgium	(13)	AHDT01000000	AHDT01000022	288,640
MC67	*B. weihenstephanensis,* isolated from soil in Denmark	(14)	AHEN01000000	AHEN01000048	56,684
MC118	*B. weihenstephanensis,* isolated from soil in Denmark	(14)	AHEM01000000	AHEM01000066	26,595

### Sequence diversity of the *ces* gene cluster

All the emetic strains harbor the seven *ces* genes with the same sizes. The two "*cereus*" isolates, IS075 and AH187, only share three nucleotide variances for their *cesB* gene. For the five "*weihenstephanensis*" isolates, MC67 and MC118 from Denmark display only one synonymous mutation, in *cesA* and in *cesT*, respectively, and CER057, CER074 and BtB2-4 from Belgium are 100% identical. Each *ces* gene displays 90 ~ 95% identity between *B. cereus* and *B. weihenstephanensis*, and 95 ~ 100% identity within *B. weihenstephanensis* isolates. Similar but slightly lower identity levels were observed for the corresponding proteins. Thus, based on the concatenated *ces* genes and protein sequences, two main clusters, namely "*cereus*" and "*weihenstephanensis*", could be distinguished, and within "*weihenstephanensis*" cluster, two subsequent clades were identified (Figure 1B).

### Genomic location of the *ces* gene clusters

**Figure 2:**
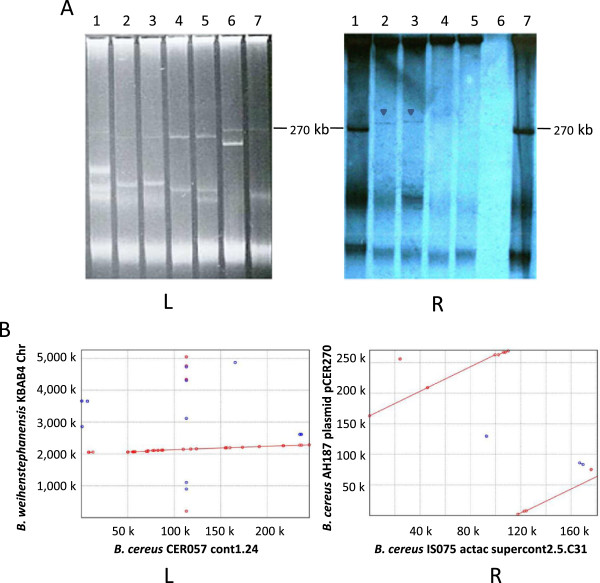
**Genomic location of the cereulide gene cluster. (A)** Genomic location of the cereulide gene cluster of emetic *B. cereus* group isolates determined by plasmid profiling (L) and hybridization (R). Lane 1: IS075, lane 2: MC118, lane 3: MC67, lane 4: CER057, lane 5: BtB2-4, lane 6: non cereulide-producing *B. cereus* isolate CER071, lane 7: AH187. The probe used was *cesB* internal fragment amplified with EmF and EmR primers from the reference strain AH187. pMC118 and pMC67, displaying a larger size than pCER270, are indicated by a dark triangle. **(B)** Linear arrangement of the contig containing the *ces* gene cluster of (L) CER057 with the chromosome of KBAB4 and (R) IS075 with the plasmid pCER270. Aligned segments are represented as dots (20 ~ 65 bp) and lines (>65 bp), with red and blue colors refer to forward and reverse matching substrings, respectively.

IS075 harbors a larger plasmid pool than AH187. The cereulide gene cluster of IS075 was observed to be located on a large plasmid with a size similar to that of pCER270 (270 kb) in AH187 (Figure 2A). Like pCER270, IS075 was PCR-positive to the pXO1 backbone genes pXO1-11, pXO1-14, pXO1-45, pXO1-50 and pXO1-55, which all encode hypothetical proteins (data not shown). It was also observed that the IS075 contig containing the *ces* gene cluster is ca. 180.7 kb with 146 predicted CDSs, of which 85.6% matched to those of pCER270, with a good synteny (Figure 2B). This indicated that the emetic plasmid in IS075 is pXO1-like with high similarity to pCER270. The deduced proteins from 21 predicted CDSs not matching those of pCER270 were blasted with databases (Nr and Swissprot). The result showed that two matched putative transposases, one was related to putative DNA topoisomerases I, one to putative transcriptional repressors, and the others to hypothetical proteins, all with homologs in other *B. cereus* group plasmids.

For BtB2-4 and CER057, although large plasmid with smaller size to pCER270 was observed in the profile, no hybridization signal was detected (Figure 2A). It was observed that the contig containing the *ces* gene cluster in CER057 is about 245.4 kb with 215 predicted CDSs, of which 80% and 85% matched those of the chromosomes of AH187 and KBAB4, respectively. Except for the *ces* genes, the deduced proteins of 25 predicted CDSs not matching the chromosome of KBAB4 were compared to protein databases (Nr and Swissprot). It was found that four CDSs encode putative transposase, acetyltransferase, phage integrase, and phosphoglycolate phosphatase, 17 encode hypothetical proteins with chromosomal homologs among *B. cereus* group strains and four had no hit. The linear alignment showed that the main matches were located in chromosome positions 2.15 M ~ 2.34 M for AH187, and 2.05 M ~ 2.28 M for KBAB4 (Figure 2B). Thus, it is most likely that the *ces* gene cluster in CER057 has a chromosomal location.

The hybridization bands of MC118 and MC67 are larger than that of pCER270, although the corresponding plasmid bands are rather weak (Figure 2A). This strongly suggests that the cereulide genetic determinants of both MC118 and MC67 (named pMC118 and pMC67) are located on plasmids larger than pCER270, which were PCR-negative to pXO1 backbone genes. Unfortunately, the contigs containing the *ces* gene clusters in MC67 and MC118 were very short, ca. 56.7 and 26.6 kb, respectively*.* Besides the seven *ces* genes, 30 putative CDSs were predicted in the larger contig of MC67, of which 9 had no hit, and the other 21 had homologs in the plasmids or chromosomes of other *B. cereus* group strains, including putative transposases, spore germination proteins, thiol-activated cytolysin, dehydratase and hypothetical proteins. However, although the gapped genome of MC67 was tentatively aligned with all the published plasmid sequences of the *B. cereus* group using the MAUVE contig aligner, no obvious colinear match was observed to large fragment (data not shown).

### Identification of putative mobile genetic elements (MGEs) flanking the cereulide genetic determinants

About 5 kb DNA sequences upstream of *cesH* and downstream of *cesD* from the "*ces*" contigs were used for detailed analysis. In the case of MC67 and MC118, because the available flanking sequences were shorter they were obtained by primer walking.

**Figure 3:**
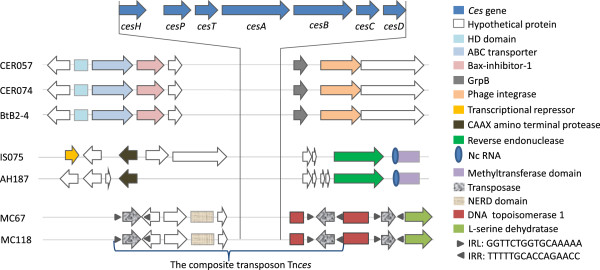
**Physical map of the sequences flanking the emetic gene clusters.** About 5 kb DNA sequences upstream of *cesH* and downstream of *cesD* were analyzed for CER057, CER074, BtB2-4, IS075 and F4810/75, respectively, and due to the available sequences are shorter, about 5 kb DNA sequences upstream of *cesH* and 2.2 kb downstream of *cesD* were analyzed for MC67 and MC118. The composite transposon Tn*ces* in emetic *B. weihenstephanensis* MC67 and MC118 is indicated by black triangles. The Tn*ces* consists of *ces* gene cluster flanked by two copies of IS element at each end in the opposite direction, containing a transposase gene and 16 bp invert repeats (IRL and IRR) at both ends. Sign and color codes are indicated on the right hand side. Physical map is not at scale.

Three types of flanking sequences could be observed (Figure 3). A potential group II intron, carrying an ncRNA and reverse endonuclease gene, is located 2.4 kb downstream of *cesD* in the plasmid of both AH187 and IS075, while an integrase/recombinase gene is located 1.1 kb downstream of *cesD* in chromosome of BtB2-4, CER057 and CER074. No other potential MGEs were observed in the flanking sequences of *cesH* of these strains. Strikingly, the *ces* gene cluster of pMC67 and pMC118 was found to be flanked by two copies of an IS element at each end, in opposite orientation (located ca. 2 kb from *cesH* and 800 bp from *cesD*), reminiscent of a typical class I composite transposon (designated Tn*ces*). This IS element (named IS*ces*) is 853 bp, contains a transposase gene and 16 bp terminal invert repeats (IR) and belongs to the IS*6* family. In addition, an NERD domain or topoisomerase domains, belonging to DNA-breaking-rejoining enzyme superfamily, were also observed located between IS*ces* and *cesH* and downstream of *cesD* and IS*ces* on pMC67 and pMC118, respectively*.* Downstream of the Tn*ces*, there is another transposase-encoding ORF showing high identity with the upstream ones, but with a shorter size. It is also flanked by the 16 bp IR (Figure 3).

### Transposition of IS*ces*-based composite transposon

In order to test the potential "transposability" of Tn*ces*, the *ces* gene cluster was replaced by a Km^R^ gene marker and a recombinant plasmid pTn*km* was created and used for the transposition assay using a well-developed mating-out assay [32,33]. Conjugation between the donor strain *E. coli* JM109 (R388, pTn*km*) and the recipient strain HB101 (Sm^R^) was performed. The average transposition frequency of Tn*ces::km* onto R388 in three independent experiments was estimated as 2.31 × 10^-3^ (number of Km^R^Tp^R^Sm^R^ transconjugants per Tp^R^Sm^R^ transconjugants). The final transfer frequency, which is equal to the actual transposition frequency multiplied by the conjugation frequency, was calculated as 1.04 × 10^-3^ Km^R^Sm^R^ transconjugants per Sm^R^ recipient. 60 transconjugants were randomly screened for Ampicilin resistance by disk diffusion assays and all displayed a positive result, indicating the formation of a cointegrate between the host chromosome and pTn*km*.

**Figure 4:**
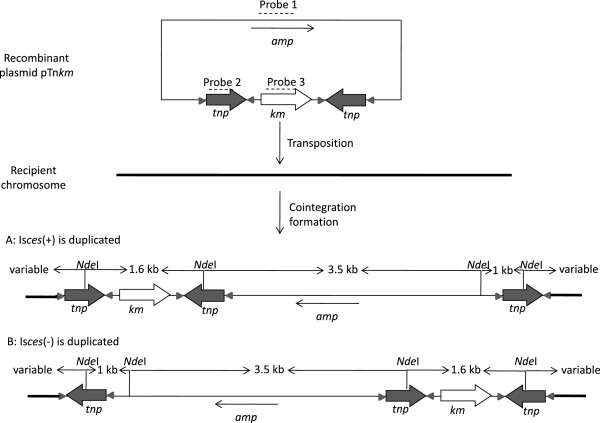
**Sketch drawing of the replicative transposition of Tn*ces*::*km *into recipient chromosome and the strategy of hybridization.** The transposase-mediated fusion of pTn*km* and the target molecules generate a third copy of IS*ces*. There are two theoretically possible results of transposition, depending on which IS*ces* is duplicated. Three probes 1, 2, and 3, indicated by dotted lines, represent an internal fragment of *bla* in cloning vector pUC18, IS*ces*, and *Km*, respectively, were used for the survey of the transposition. The *Nde*I sites in km^R^sm^R^ transconjugants were indicated. No matter which IS*ces* was duplicated, hybridization with probe 1 and 3, a 3.5 kb band and a 1.6 kb band is expected, respectively; with probe 2, besides the 1 kb and 3.5 kb expected bands, extra bands with variable sizes in each independent transconjugant are probably detected due to multi-transpositions. Although there is also a (remote) possibility for the duplication of the whole Tn*ces*::*km* element, the result will be similar except that more bands with probe 2 are expected.

**Figure 5:**
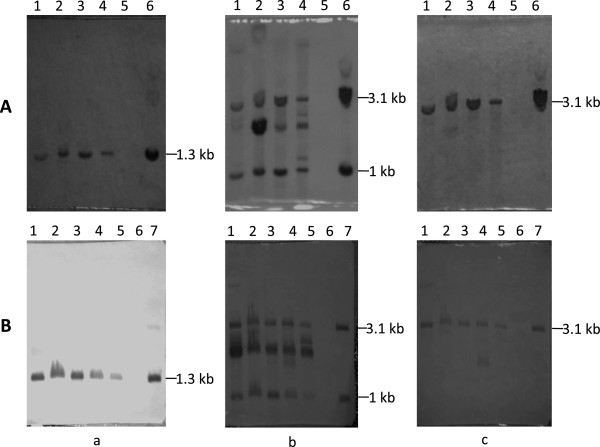
**Southern blot hybridization analysis of the transconjugants of Tn *ces *::*km *transposition in *E. coli *HB101.** Two independent hybridizations were performed. **A**: lane 1–4, independent Km^R^Sm^R^ transconjugants, lane 5, HB101, lane 6, JM109 (pTn*km*); and **B**: lane 1–5, independent Km^R^Sm^R^ transconjugants, lane 6, HB101, lane 7, JM109 (pTn*km*). Three probes of *Km* (a), IS*ces* (b) and *blapuc18* (c), respectively were used for hybridization as illustrated in Figure 4.

In order to distinguish whether the Km^R^Sm^R^ transconjugants were achieved by transposition or other recombination events leading to plasmid integration, and whether the transposition happened randomly, a Southern-blot analysis was performed on nine transconjugants from two independent conjugation experiments that were randomly selected according to the resistance screening and the PCR validation. The hybridization was conducted on the transconjugants *Nde*I-digested genomic DNA using an internal *bla* fragment (pUC18), IS*ces* and *km* as probes (Figure 4). Both hybridizations with the *bla* and *km* probes produced a single signal band, the former confirming the formation of a cointegrate of the whole pTn*km* into the recipient chromosome. Using the IS*ces* probes, besides the expected 1 and 3.1 kb bands observed in all the transconjugants, at least one extra band with variable sizes was observed in the nine tested transconjugants, indicating that independent multi-events had occurred at distinct genomic sites (Figure 5).

To detect if the transposition of Tn*ces::Km* displayed target site biases, the flanking sequences of insertion sites of the transconjugants used in hybridization were determined by primer walking. For three transconjugants, it was found that Tn*ces::Km* insertions occurred in three distinct sites on plasmid R388 and that an 8-bp direct repeat (DR) was produced after transposition (Table 2), which is a typical feature of IS*6* family members (see the ISfinder database, http://www-is.biotoul.fr) [34]. For the other six transconjugants, although repeated several times, it is difficult to get the flanking sequences of insertion sites by primer walking, probably due to sequence complexity caused by multiple transposition events of IS*ces.*

**Table 2:** **DNA sequences flanking the insertion sites after random transposition of IS** ***ces *** **based transposon Tn *ces *::** ***km *** **onto R388**

**Transconjugants**	**Sequence of insertion sites (5’ to 3’)**
Tn02	GCCAACTTCCAAAGGAAAGAAGCC*GCATAACC*-IS*ces*-*GCATAACC*TGCCCTCCCCCGCTCCGGCGGGGG
Tn04	GAAGGCCAACGGTGGCGCCCAAGA*AGGATTTC*-IS*ces*-*AGGATTTC*CGCGACACCGAGACCAATAGCGGAA
Tn05	GAGCGGGCTTTTTTATCCCCGGAAG*CCTGTGGA*-IS*ces*-*CCTGTGGA*TAGAGGGTAGTTATCCACGTGAAAC

The underlined sequences refer to the duplicated target sequences (DR).

## Discussion

The taxonomy of *B. cereus* group has long been controversial, since many of the species are genetically heterogenous, with the exception of *B. anthracis,* which is essentially a clone in nature [35]. One of the reasons of this difficulty is that many toxins used for classification are encoded on MGEs that have HGT potential, e.g. plasmids or transposons [3,36,37]. Cereulide may cause severe and potential lethal infection during an "emetic" form of *B. cereus* food poisoning. Most emetic *B. cereus* strains belong to a homogeneous group of *B. cereus sensu stricto.* Although rare, the emetic *B. weihenstephanensis* strains were recently isolated in nature [13]. Furthermore, a heat stable toxin, structural related to cereulide, has also been found in *Paenibacillus tundra* strain [38]. As a consequence, the intra- and inter-species diversity and potential transmission of the cereulide biosynthetic gene cluster is therefore thought provoking.

In this study, the sequence diversity of emetic *B. cereus sensu stricto* and *B. weihenstephanensis* was analyzed. Since emetic *B. cereus sensu stricto* had been found to be restricted to a homogeneous group [30], only two *B. cereus sensu stricto* isolates were analyzed and compared the other five known *B. weihenstephanensis*. Except for AH187, the unfinished gapped genome sequences of the other emetic isolates were recently submitted [39]. As expected, the two emetic *B. cereus sensu stricto* isolates share very similar gene content in genome level. Furthermore, their "*ces*" plasmids are quite coherent in terms of synteny, protein similarity and gene content. Compared to AH187, IS075 has a larger plasmid pool, of which the "*ces*" plasmid is pXO1-like, but the presence of a pXO2-like plasmid was also indicated [40].

Sequence diversity between *B. cereus sensu stricto* and *B. weihenstephanensis* or within *B. weihenstephanensis* was observed. It was also evidenced that the *ces* cluster had undergone horizontal gene transfer (HGT). This could be clued by the fact that the cluster is present in different hosts (*B. cereus sensu stricto vs. B. weihenstephanensis*), which have different chromosomal background, and displays different genomic locations (plasmids *vs.* chromosome). Moreover, another striking indication for HGT was the presence of putative MGEs in all tested emetic strains.

The composite transposon, Tn*ces*, located on large plasmids (pMC67/pMC118) in two *B. weihenstephanensis* strains isolated from soil in Denmark was identified. The mobility of Tn*ces* was also proved by transposition experiments performed on a Tn*ces*-derived element, indicating a HGT potential of the cereulide gene cluster in pMC67/pMC118. Although the *ces* gene cluster is not flanked by IS elements in the other two types of emetic isolates, a Group II intron carrying an endonuclease gene in AH187 and IS075, and a putative integrase/recombinase gene in CER057, CER074 and BtB2-4 were also observed downstream of *cesD*. Both Group II intron and recombinase can potentially be involved in genome dynamics. Group II introns are self-splicing mobile retroelements, some of which have been shown experimentally to be able to invade new DNA sites and transfer between species, sometimes accompanied by adjacent sequence deletion or rearrangement [41-43]. This also relates to previous observations that bacterial group II introns tend to be located within mobile DNA elements such as plasmids, IS elements, transposons or pathogenicity islands (PAI), which could account for their spread among bacteria [44-46].

Based on our results, it is reasonable to suggest that MGEs have played a key role in the transmission of the cereulide gene cluster. In many cases, plasmids encode passenger genes originated *via* HGT that generally confer adaptive functions to the host cell, the classic example being antibiotic resistance genes. For instance, the NRPS gene cluster responsible for the production of β-lactam antibiotics (e.g. penicillins and cephalosporins) was proved to be transmitted by HGT from bacteria to bacteria and from bacteria to fungi [47,48]. This is also the general mode for toxin evolution [49,50]. In contrast, as a natural analog, a recent study reported that a vertical transmission (VT) origin rather than a HGT for the *vlm* gene cluster in *Streptomyces* spp. Although there is a significant structure and toxicology similarity between valinomycin and cereulide and an organizational similarity between the *vlm* gene cluster and the *ces* gene cluster, they are highly divergent from each other at the DNA level [51]. They may also have quite different evolution history.

The conjugative and transfer promoting capacities of the emetic plasmids were also assessed by bi- and tri-parental matings, respectively. None were indicative of self-conjugative or mobilizable activities, at least under the conditions used in the assay (detection limit of 10^-7^ T/R) (data not shown). Yet, the emetic strains can host the conjugative plasmid pXO16, which could be transferred from its native *B. thuringiensis* sv. *israelensis* to the emetic strains and, subsequently from the emetic strains to the original *B. thuringiensis* sv. *israelensis* host [52].

An important concern arising from this study is that the cereulide gene cluster may have the potential to be transmitted by transposition and, therefore, if the emetic strain can randomly encounter the conjugative plasmid pXO16 in nature, transposition of the cereulide gene cluster into pXO16 might happen at a low frequency, and as a consequence the resulting emetic pXO16, crossing boundaries within the *B. cereus* group by conjugation, could pose a serious public health issue.

## Conclusion

Emetic *B. cereus* group isolates display more variations than originally thought. The cereulide biosynthesis gene cluster was present in different hosts (*B. cereus sensu stricto* and *B. weihenstephanensis*), which have different chromosomal background and display different genomic locations (plasmids *vs.* chromosome). The sequences of cereulide genetic determinants are diverse and coevolved with the host. Three types of MGEs were identified in the flanking sequences of the cereulide biosynthesis gene cluster, of which the transposition capacity of a resistant derivative of the composite transposon Tn*ces* in *E. coli* was demonstrated. Further study is needed to look for appropriate genetic tools to analysis the transposition of Tn*ces* in *Bacillus* spp. and the dynamics of other MGEs flanking the *ces* gene clusters.

## Methods

### Strains and plasmids

Emetic strains used in this study are listed in Table 1. A non cereulide-producing *B. cereus* isolate CER071 was used as negative control. *E. coli* DH5α and JM109 were used as bacterial hosts in electroporation experiments. Plasmid R388 (Trimethoprim resistant) [53], a conjugative plasmid devoid of transposon, was used for transposition assay. *E. coli* was routinely cultivated at 37°C in Luria-Bertani (LB) media. *B. cereus* group strains were grown at 30°C. Antibiotics were used at the following concentrations: Kanamycin (Km), 50 μg/ml; Ampicilin (Amp), 50 μg/ml and Trimethoprim (Tp), 50 μg/ml.

### Insertion site determination of the cereulide gene cluster and Tn*ces::Km*

Regions flanking the cereulide gene cluster sites of the emetic *B. cereus* isolates and the target site and flanking sequences of the composite transposon were obtained by the method of genome walking (Takara genome walking kit), using the primer walking sets listed in Table 3. All the sequences obtained by this method were validated by PCR and subsequent sequencing.

**Table 3:** Primers used in this study

**Primers**	**Target**	**Sequences (5’ → 3’)**
EmF	*cesB*	GACAAGAGAAATTTCTACGAGCAAGTACAAT
EmR		GCAGCCTTCCAATTACTCCTTCTGCCACAGT
14 F	pXO1-14	GGTAAAGAGTGCGGAAAATGA
14R		AATACGCCAACGCCAACTTA
45 F	pXO1-45	TGCAGCTCGTAATCCACAG
45R		TGCTAATGATAAAACGCCTGG
50 F	pXO1-50	TTCGTACAGATGAAACACAGG
50R		GTGCCTCAAGATGAACCTTC
55 F	pXO1-55	GATAGAGACTGCTCTTGGGAA
55R		GGTCTTAGCCATGAGAGTAAAAACA
58 F	pXO1-58	TGTGATGGACCTTTGTATTAATTTGT
58R		ATACCCCGCATGGAGCTTAG
ISF_*Sac*I	IS*ces*	GCA**GAGCTC**GGTTCTGGTGCAAAAACTTCAGGACA
ISR_*Xba*I		GCA**TCTAGA**GGTTCTGGTGCAAAAAGATAATAAAG
ISF_*Hin*dIII	IS*ces*	GCA**AAGCTT**GGTTCTGGTGCAAAAACTTCAGGACA
ISR_*Bam*HI		GCA**GGATCC**GGTTCTGGTGCAAAAAGATAATAAAG
KmF_*Xba*I	*Km*	TCA**TCTAGA**TAAACCCAGCGAACCATTTG
KmR_*Bam*HI		TCA**GGATCC**TCTAGGTACTAAAACAATTCATCCAG
ISF3	IS*ces*	TCTGGTGCAAAAACTTCAGG
ISR3		AAGTCGCATACGACCAGGTA
kmF3	*Km*	GAGGATGAGGAGGCAGATTG
KmR3		CGGCCAGATCGTTATTCAGT
APF1	*bla*	TTTGCCTTCCTGTTTTTGCT
APR1		TTGCCGGGAAGCTAGAGTAA
ISL-SP1		CTTCATCCTCTTCGTCTTGGTAGC
ISL-SP2		GGTTCGCTGGGTTTATCTAGAGGT
ISL-SP3		GACAGACTGGTCCCGTAAATCAAC
ISR-SP1		ATATCGGGGAAGAACAGTATGTCG
ISR-SP2		GTACCTAGAGGATCCGGTTCTGGT
ISR-SP3		GACAGACTGGTCCCGTAAATCAAC
IS-LR		CTTTCGAATCAACAGCACGA
CesD-SP1		GGCCTATTGTATAATGACAACG
CesD-SP2		GGTGTATTATTTATCTTCGCCTG
CesD-SP3		GGTATTTTAGGGGCGAAGGTTC
MH-SP1		CACTCTTGCGTTTTTGCGTATC
MH-SP2		AAACAATGAGCCCACCCCGAAA
MH-SP3		CGCTTTTCCACATTCTTTACGG

### DNA manipulation and plasmid construction

Plasmid and genomic DNA were isolated using Plasmid Mini-Midi kits and Bacterial genome extraction kit (QIAGEN), respectively. Primers (Table 3) were designed to amplify and sequence *ces* gene fragments based on pCER270 sequence [GenBank: NC_010924]. Standard PCR amplification experiments were performed with primers listed in Table 3. In order to evaluate the possible transposition capacity of the composite transposon containing the cereulide gene cluster of MC118, a composite transposon Tn*ces::Km* was constructed by the replacement of the cereulide gene cluster with the Km^R^ marker as follows. A 1.3 kb fragment containing the Km^R^ gene was amplified with the primer pair KmF_*Xba*I/KmR_*Bam*HI. Two 853 bp IS*ces* elements (see below) containing a transposase gene, flanked by the left- and right IR, were amplified with the primer pairs ISF_ *Sac*I/ ISR_*Xba*I and ISF_ *Hin*dIII/ ISR_*Bam*HI. Products were digested with the appropriate enzymes, and mixed in a four-way ligation with *Bam*HI-*Xba*I-cleaved Km^R^ fragment, and *Sac*I-*Hin*dIII-cleaved pUC18 vector, pTn*Km* was created to carry Tn*ces::km* with two copies of IS*ces* element in opposite orientations flanking the Km^R^ marker. The electroporation of recombinant plasmid into *E. coli* DH5a and JM109 was as described by Sambrook and coll. [54].

### Plasmid profiling and hybridization

Plasmid profiling of the emetic isolates was performed according to Andrup *et al*. [55]. Genomic DNA from *E. coli* strains HB101, JM109 (pTn*Km*), JM109 (R388, pTn*Km*) and transconjugants were digested with *Nde*I and run in a 0.8% agarose gel electrophoresis before the separated DNA fragments were transferred from agarose gels to a positively charged nylon membrane (Boehringer Mannheim, Germany). DIG-labeled probes were designed by using the "PCR DIG Probe Synthesis Kit" from Roche. Probe P_ces_, consisting of an internal fragment of *cesB* using EmF and EmR primers, was used for the location of cereulide gene cluster. Probes 1, 2, and 3, which consisted of an internal fragment of *bla*_*pUC18*_ using APF1 and APR1 primers, an internal fragment of IS using ISF3 and ISR3 primers, and an internal fragment of *km* using kmF3 and KmR3 primers, were used for transposition survey. After transfer and fixation of the DNA on the membrane, the hybridization was performed with the "DIG High Prime DNA Labeling and Detection Starter Kit I" (Roche Diagnostic, Mannheim, Germany), according to the manufacturer’s instructions.

### Transposition experiments

The transposition of the pTn*Km* was examined using a mating-out experiment, as previously described [32,33]. For this purpose, *E. coli* JM109 harboring pTn*Km* and plasmid R388 (Tp^R^) was used as the donor to mate with *E. coli* HB101 (Sm^R^) on a membrane filter. The transposition frequency was expressed as the number of Km^R^Sm^R^ transconjugants per Sm^R^ recipients (T/R) and the plasmids in the transconjugants were further characterized by PCR and restriction digestion.

### Sequence analysis

The complete genome sequence of AH187 and the gapped genome sequences of the other six emetic strains were obtained from NCBI (Table 1). A fragmented all-against-all comparison analysis was performed using Gegenees (version 1.1.5) software [31] by fragmenting genomes and comparing all pieces with all emetic genomes and *B. cereus* ATCC 10987 [GenBank: NC_ 003909], ATCC 14579 [GenBank: NC_ 004722] and *B. weihenstephanensis* KBAB4 [GenBank: NC_010184]. The heat-plot is based on a fragmented alignment using BLASTN made with settings 200/100. The cutoff threshold for non-conserved material was 30%. Based on this all-against-all approach, a corresponding phylogenetic dataset can be extracted and then a tree was constructed using neighbor joining method by splitstree4 (version 4.12.8) with this dataset.

Each *ces* gene and the concatenated sequences, as well as the deduced amino acid sequences, were aligned by MEGA version 5.2 software. A neighbor-joining (NJ) phylogenetic tree based on the concatenated gene sequences was constructed with a bootstrap of 1,000.

The contigs containing the *ces* gene cluster were compared with the genomes of AH187 and *B. weihenstephanensis* KBAB4 by BLASTN with an e-value cutoff of 1e-5. Linear alignment was finished by MUMmer software package (release 3.23) [56]. The sequences upstream of *cesH* and downstream of *cesD* were obtained from the complete genome sequence of AH187 and the contigs with the *ces* gene cluster located within the gapped genome sequences of the emetic strains (NCBI - Table 1), except that MC67 and MC118 by primer walking [GenBank: KF554002, KF554003, KF554006, KF554007].

## Competing interests

The authors declare that they have no competing interests.

## Authors’ contributions

XM carried out the mating-out and transposition experiments, and wrote the paper; KX performed the bioinformatics analysis; LY carried out primer walking and partial sequencing; ZY revised the paper; XH designed the study, constructed the recombinant plasmid, analyzed the data and wrote the paper; JM designed the study, analyzed data and revised the paper. All authors read and approved the final manuscript.
